# Supplementary material for: Numerous cultivated and uncultivated viruses encode ribosomal proteins
Source: Nat Commun. 2019 Feb 14;10:752. doi: 10.1038/s41467-019-08672-6 (PMC6375957; doi:10.1038/s41467-019-08672-6)
Supplement: Supplementary file 1 — Supplementary Information [file 41467_2019_8672_MOESM1_ESM.pdf]

SUPPLEMENTARY INFORMATION

Numerous cultivated and uncultivated viruses encode ribosomal proteins

Carolina M. Mizuno[1#], Charlotte Guyomar[2#], Simon Roux[3], Régis Lavigne[4], Francisco Rodriguez-Valera[5], Matthew B. Sullivan[6,7], Reynald Gillet[2], Patrick Forterre[1], Mart Krupovic[1*]

[1] – Unité de Biologie Moléculaire du Gène chez les Extrêmophiles, Département de Microbiologie, Institut Pasteur, Paris, France

[2] – Université Rennes, CNRS, UBL, Institut de Génétique et Développement de Rennes (IGDR) - UMR 6290 -F35042 Rennes France

[3] – Department of Energy Joint Genome Institute, Walnut Creek, CA 94598, USA

[4] – Inserm U1085 IRSET, Université de Rennes 1, 35000 Rennes, France; Protim, 35042 Rennes, France.

[5] – Departamento de Producción Vegetal y Microbiología, Evolutionary Genomics Group, Universidad Miguel Hernandez, Alicante, Spain

[6] – Department of Microbiology, The Ohio State University, Columbus, OH 43210, United States

[7] – Department of Civil, Environmental and Geodetic Engineering, The Ohio State University, Columbus, OH 43210, United States

# – These authors contributed equally.
* – To whom correspondence should be addressed. Email: krupovic@pasteur.fr

Supplementary Table 1. List and PFAM accessions of ribosomal protein domains searched for in viral proteomes.

Domain ID	Length (aa)	Accession	Description
Img2	87	PF05046.9	Mitochondrial large subunit ribosomal protein (Img2)
L31	103	PF09784.4	Mitochondrial ribosomal protein L31
L51_S25_CI-B8	52	PF05047.11	Mitochondrial ribosomal protein L51 / S25 / CI-B8 domain
Mit_ribos_Mrp51	315	PF11709.3	Mitochondrial ribosomal protein subunit
Mitoc_L55	116	PF09776.4	Mitochondrial ribosomal protein L55
MRL1	134	PF13003.2	Ribosomal protein L1
MRP_L53	51	PF10780.4	39S ribosomal protein L53/MRP-L53
MRP-L20	164	PF12824.2	Mitochondrial ribosomal protein subunit L20
MRP-L27	113	PF09809.4	Mitochondrial ribosomal protein L27
MRP-L28	157	PF09812.4	Mitochondrial ribosomal protein L28
MRP-L46	111	PF11788.3	39S mitochondrial ribosomal protein L46
MRP-L47	87	PF06984.8	Mitochondrial 39-S ribosomal protein L47 (MRP-L47)
MRP-S22	243	PF10245.4	Mitochondrial 28S ribosomal protein S22
MRP-S23	128	PF10484.4	Mitochondrial ribosomal protein S23
MRP-S25	231	PF13741.1	Mitochondrial ribosomal protein S25
MRP-S27	417	PF10037.4	Mitochondrial 28S ribosomal protein S27
MRP-S32	96	PF10210.4	Mitochondrial 28S ribosomal protein S32
MRP-S35	105	PF10246.4	Mitochondrial ribosomal protein MRP-S35
PDCD9	428	PF07147.7	Mitochondrial 28S ribosomal protein S30 (PDCD9)
PSRP-3_Ycf65	49	PF04839.8	Plastid and cyanobacterial ribosomal protein (PSRP-3 / Ycf65)
Ribos_L4_asso_C	80	PF14374.1	60S ribosomal protein L4 C-terminal domain
Ribosomal_60s	88	PF00428.14	60s Acidic ribosomal protein
Ribosomal_L1	221	PF00687.16	Ribosomal protein L1p/L10e family
Ribosomal_L10	100	PF00466.15	Ribosomal protein L10
Ribosomal_L11	69	PF00298.14	Ribosomal protein L11, RNA binding domain
Ribosomal_L11_N	60	PF03946.9	Ribosomal protein L11, N-terminal domain
Ribosomal_L12	68	PF00542.14	Ribosomal protein L7/L12 C-terminal domain
Ribosomal_L13	128	PF00572.13	Ribosomal protein L13
Ribosomal_L13e	179	PF01294.13	Ribosomal protein L13e
Ribosomal_L14	122	PF00238.14	Ribosomal protein L14p/L23e
Ribosomal_L14e	77	PF01929.12	Ribosomal protein L14
Ribosomal_L16	133	PF00252.13	Ribosomal protein L16p/L10e
Ribosomal_L17	97	PF01196.14	Ribosomal protein L17
Ribosomal_L18e	129	PF00828.14	Ribosomal protein L18e/L15
Ribosomal_L19	113	PF01245.15	Ribosomal protein L19
Ribosomal_L19e	148	PF01280.15	Ribosomal protein L19e
Ribosomal_L2	77	PF00181.18	Ribosomal Proteins L2, RNA binding domain
Ribosomal_L2_C	130	PF03947.13	Ribosomal Proteins L2, C-terminal domain
Ribosomal_L20	108	PF00453.13	Ribosomal protein L20
Ribosomal_L21e	99	PF01157.13	Ribosomal protein L21e
Ribosomal_L22	105	PF00237.14	Ribosomal protein L22p/L17e
Ribosomal_L23	92	PF00276.15	Ribosomal protein L23
Ribosomal_L23eN	54	PF03939.8	Ribosomal protein L23, N-terminal domain
Ribosomal_L24e	71	PF01246.15	Ribosomal protein L24e
Ribosomal_L3	263	PF00297.17	Ribosomal protein L3
Ribosomal_L30	52	PF00327.15	Ribosomal protein L30p/L7e
Ribosomal_L31	69	PF01197.13	Ribosomal protein L31
Ribosomal_L31e	83	PF01198.14	Ribosomal protein L31e
Ribosomal_L32e	110	PF01655.13	Ribosomal protein L32
Ribosomal_L33	48	PF00471.15	Ribosomal protein L33

Ribosomal_L34	44	PF00468.12	Ribosomal protein L34
Ribosomal_L34e	94	PF01199.13	Ribosomal protein L34e
Ribosomal_L35Ae	95	PF01247.13	Ribosomal protein L35Ae
Ribosomal_L35p	61	PF01632.14	Ribosomal protein L35
Ribosomal_L36	38	PF00444.13	Ribosomal protein L36
Ribosomal_L36e	99	PF01158.13	Ribosomal protein L36e
Ribosomal_L37	85	PF08561.5	Mitochondrial ribosomal protein L37
Ribosomal_L37e	55	PF01907.14	Ribosomal protein L37e
Ribosomal_L4	192	PF00573.17	Ribosomal protein L4/L1 family
Ribosomal_L41	25	PF05162.8	Ribosomal protein L41
Ribosomal_L44	77	PF00935.14	Ribosomal protein L44
Ribosomal_L5	56	PF00281.14	Ribosomal protein L5
Ribosomal_L6	77	PF00347.18	Ribosomal protein L6
Ribosomal_L6e	108	PF01159.14	Ribosomal protein L6e
Ribosomal_L6e_N	59	PF03868.10	Ribosomal protein L6, N-terminal domain
Ribosomal_L7Ae	95	PF01248.21	Ribosomal protein L7Ae/L30e/S12e/Gadd45 family
Ribosomal_L9_C	87	PF03948.9	Ribosomal protein L9, C-terminal domain
Ribosomal_L9_N	48	PF01281.14	Ribosomal protein L9, N-terminal domain
S1	75	PF00575	S1 RNA binding domain
Ribosomal_S10	97	PF00338.17	Ribosomal protein S10p/S20e
Ribosomal_S11	110	PF00411.14	Ribosomal protein S11
Ribosomal_S12	122	PF00164.20	Ribosomal protein S12
Ribosomal_S13	106	PF00416.17	Ribosomal protein S13/S18
Ribosomal_S14	55	PF00253.16	Ribosomal protein S14p/S29e
Ribosomal_S15	83	PF00312.17	Ribosomal protein S15
Ribosomal_S16	62	PF00886.14	Ribosomal protein S16
Ribosomal_S17	69	PF00366.15	Ribosomal protein S17
Ribosomal_S18	54	PF01084.15	Ribosomal protein S18
Ribosomal_S19	81	PF00203.16	Ribosomal protein S19
Ribosomal_S19e	140	PF01090.14	Ribosomal protein S19e
Ribosomal_S2	211	PF00318.15	Ribosomal protein S2
Ribosomal_S20p	84	PF01649.13	Ribosomal protein S20
Ribosomal_S21	57	PF01165.15	Ribosomal protein S21
Ribosomal_S21e	81	PF01249.13	Ribosomal protein S21e
Ribosomal_S22	45	PF08136.6	30S ribosomal protein subunit S22 family
Ribosomal_S23p	110	PF05635.6	S23 ribosomal protein
Ribosomal_S24e	84	PF01282.14	Ribosomal protein S24e
Ribosomal_S25	105	PF03297.10	S25 ribosomal protein
Ribosomal_S26e	114	PF01283.14	Ribosomal protein S26e
Ribosomal_S27	47	PF01599.14	Ribosomal protein S27a
Ribosomal_S27e	55	PF01667.12	Ribosomal protein S27
Ribosomal_S28e	69	PF01200.13	Ribosomal protein S28e
Ribosomal_S3_C	85	PF00189.15	Ribosomal protein S3, C-terminal domain
Ribosomal_S30	59	PF04758.9	Ribosomal protein S30
Ribosomal_S30AE	94	PF02482.14	Sigma 54 modulation protein / S30EA ribosomal protein
Ribosomal_S4	94	PF00163.14	Ribosomal protein S4/S9 N-terminal domain
Ribosomal_S5	67	PF00333.15	Ribosomal protein S5, N-terminal domain
Ribosomal_S5_C	74	PF03719.10	Ribosomal protein S5, C-terminal domain
Ribosomal_S6	92	PF01250.12	Ribosomal protein S6
Ribosomal_S6e	127	PF01092.14	Ribosomal protein S6e

Ribosomal_S7	148	PF00177.16	Ribosomal protein S7p/S5e
Ribosomal_S7e	190	PF01251.13	Ribosomal protein S7e
Ribosomal_S8	129	PF00410.14	Ribosomal protein S8
Ribosomal_S8e	132	PF01201.17	Ribosomal protein S8e
Ribosomal_S9	121	PF00380.14	Ribosomal protein S9/S16
VAR1	350	PF05316.7	Mitochondrial ribosomal protein (VAR1)

Supplementary Table 2. Detection of ribosomal proteins in uncultivated viral genomes. Metagenomes were classified as “Environmental”, “Engineered”, or “Host-associated” according to the GOLD database (https://gold.jgi.doe.gov). Values of pN/pS were calculated for all ribosomal proteins in a contig covered > 10x and with at least 1 SNP detected.

**Domain**	**Protein**	**In isolates**	**Total**	**With host prediction**	**Sample type**			**pN/pS (Average)**
					**Environmental**	**Engineered**	**Host-associated**	
Ribosomal_S21	bS21	x	1310	65	1289	9	8	0.10
Ribosomal_L12	bL12	x	30	2	22		8	0.35
Ribosomal_S30AE	HPF	x	15		2		13	N/A
Ribosomal_L31	bL31		15		15			0.14
Ribosomal_L33	bL33		12	3	12			0.09
Ribosomal_S9	uS9		6				6	0.08
Ribosomal_S6	bS6		5				5	N/A
Ribosomal_L11_N	uL11		3		2		1	0
Ribosomal_S15	uS15		3				3	N/A
Ribosomal_L19	bL19		3				3	0
Ribosomal_S20p	bS20		2			1	1	N/A
Ribosomal_L10	uL10		1		1			0.23
Ribosomal_L2	uL2		1		1			0.14

HPF, ribosome hibernation promoting factor.

Supplementary Table 3. Fraction of ribosomal proteins encoded within predicted temperate viruses.

**Protein**	**Total**	**# predicted temperate**	**Ratio**
Ribosomal_S21	1310	2	0,15%
Ribosomal_L12	30	2	6,67%
Ribosomal_S30AE	15	1	6,67%
Ribosomal_L31	15		0,00%
Ribosomal_L33	12		0,00%
Ribosomal_S9	6	1	16,67%
Ribosomal_S6	5	2	40,00%
Ribosomal_L11_N	3		0,00%
Ribosomal_S15	3	1	33,33%
Ribosomal_L19	3	2	66,67%
Ribosomal_S20p	2	2	100,00%
Ribosomal_L2	1		0,00%
Ribosomal_L10	1	1	100,00%

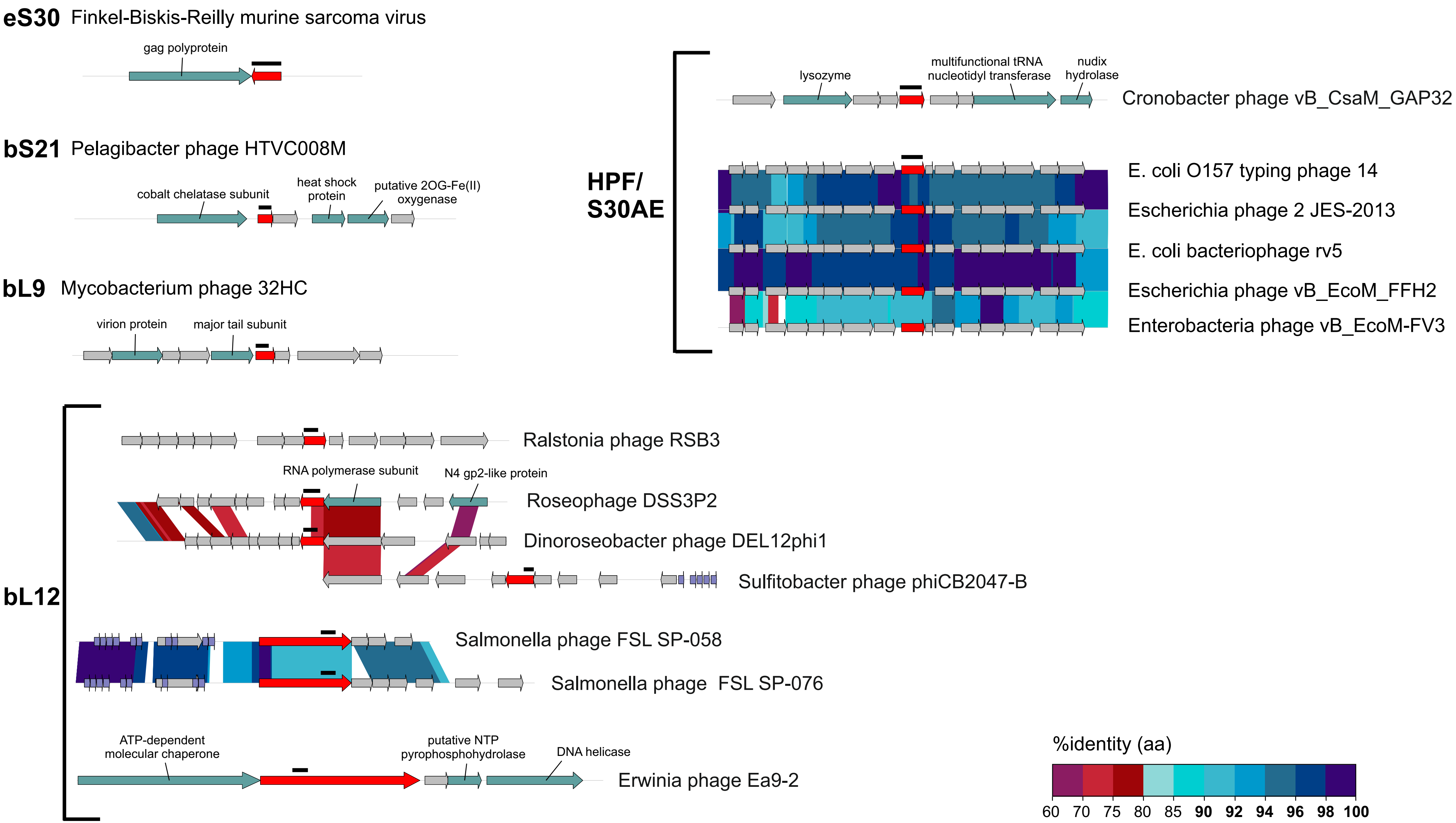

Supplementary Figure 1. Genomic context of the ribosomal protein genes identified in viruses. Genes coding for ribosomal protein domains are shown in red. The exact positions of the domains are marked by a black line on top of the gene. tRNAs are colored in purple. All comparisons were done at the amino acid level. A color scale for the % identity is shown at the bottom right.

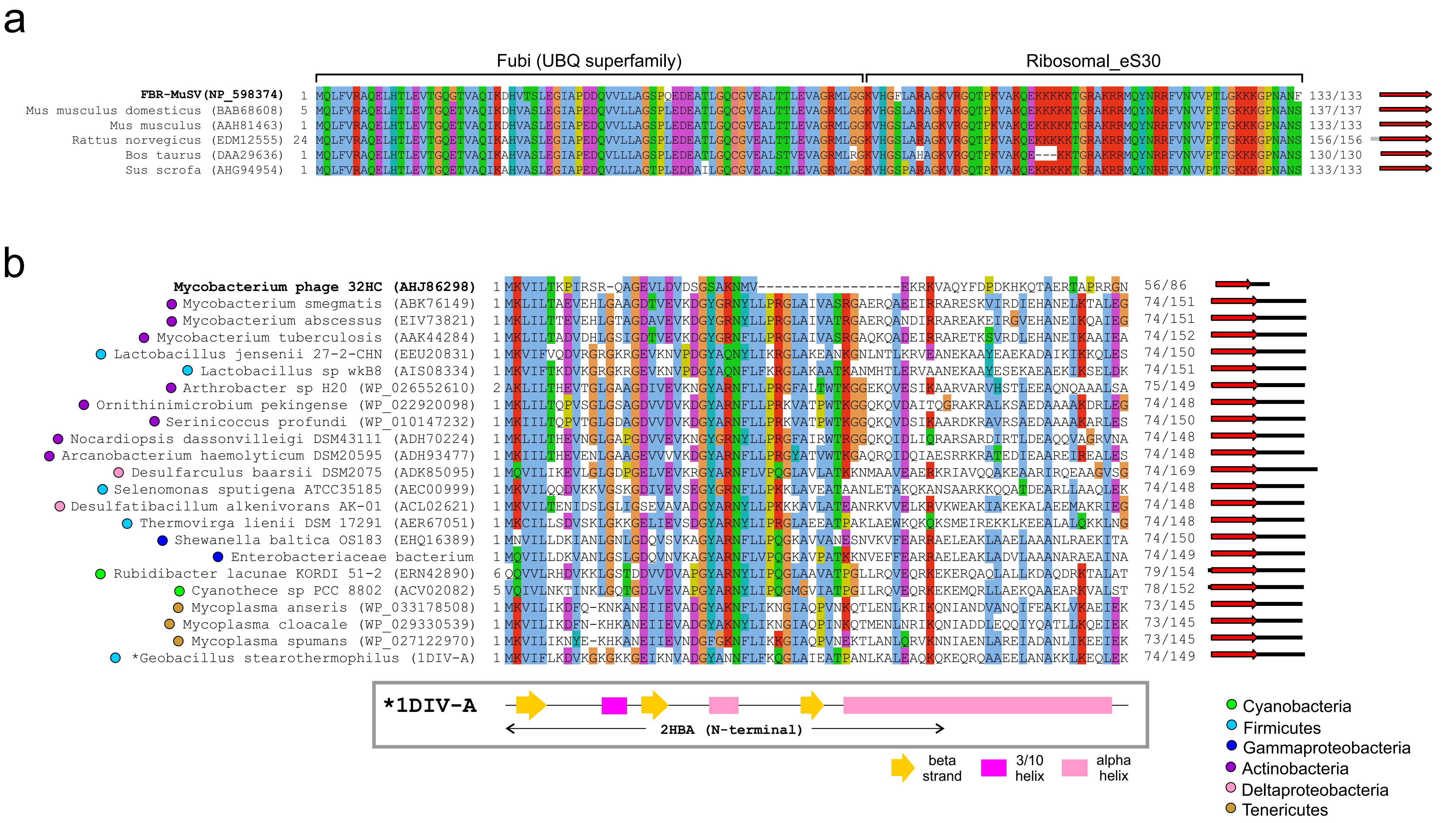

Supplementary Figure 2. Multiple sequence alignment of the phage-encoded ribosomal proteins eS30 **(a)** and bL9 **(b)** with homologous sequences encoded by cellular organisms. Taxonomic affiliations are represented by colored circles (see legend at the bottom right). Positions of the ribosomal protein domains (red arrows) within the full proteins (black lines) are shown at the right side of the alignment.

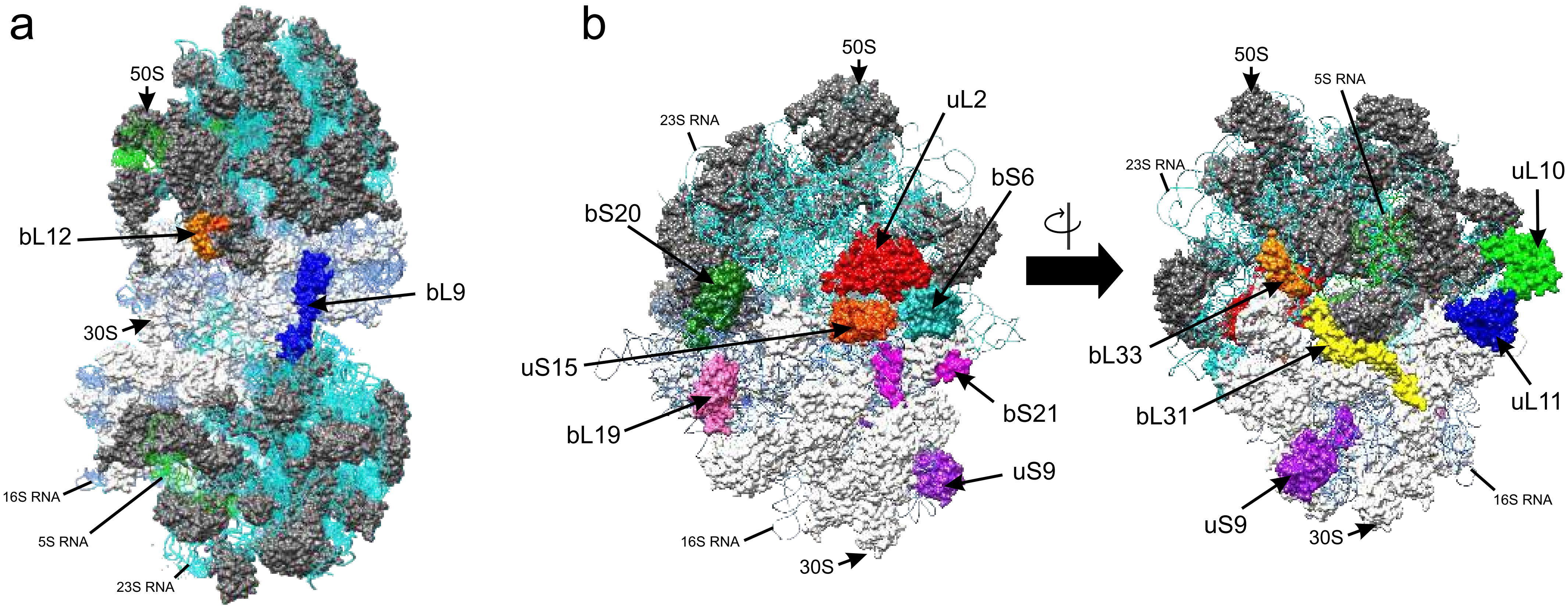

Supplementary Figure 3. Localization of virus-encoded proteins in the context of the 70S ribosome. a) Structure of the *Thermus thermophilus* ribosome (PDB id: 4V6F). b) Structure of the *E. coli* ribosome (PDB id: 5AFI). The 30S subunit is colored white and the 50S is in dark grey. Ribosomal RNAs are shown as ribbons (16S: blue, 23S: cyan, 5S: green). Ribosomal proteins found in viruses and environmental virus contigs are colored and indicated with arrows. The HPF (*Ribosomal_S30AE*) is not present in the depicted structures

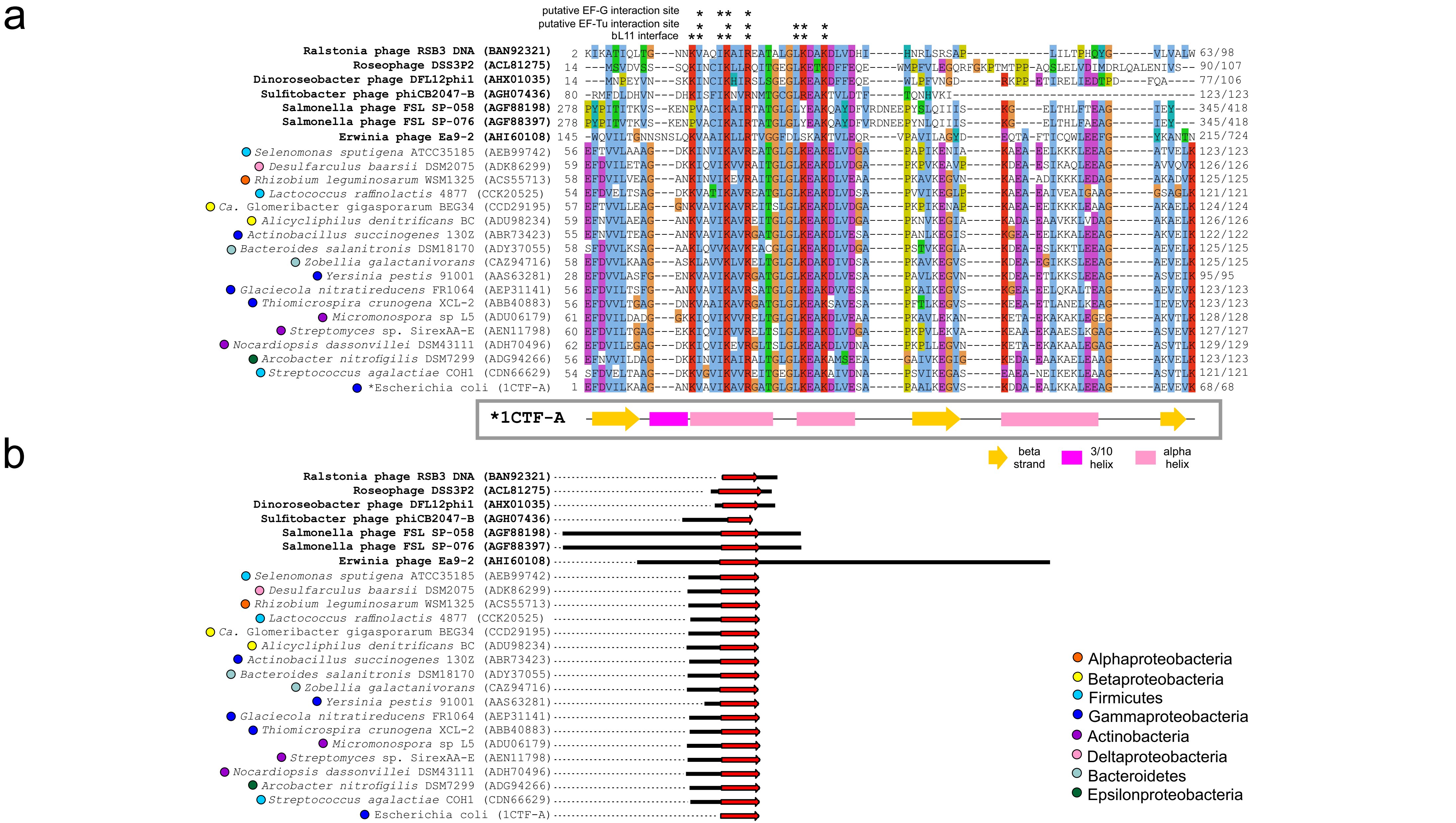

Supplementary Figure 4. a) Multiple sequence alignment of ribosomal protein bL12 identified in seven distinct phage genomes with cellular homologs. Conserved residues involved in the interaction with bL11 and elongation factors EF-G and EF-Tu are indicated by (*). **b)** Positions of the ribosomal protein domains (red arrows) within the full proteins (black line) are indicated. Taxonomic affiliations are represented by colored circles (see legend at the bottom right).

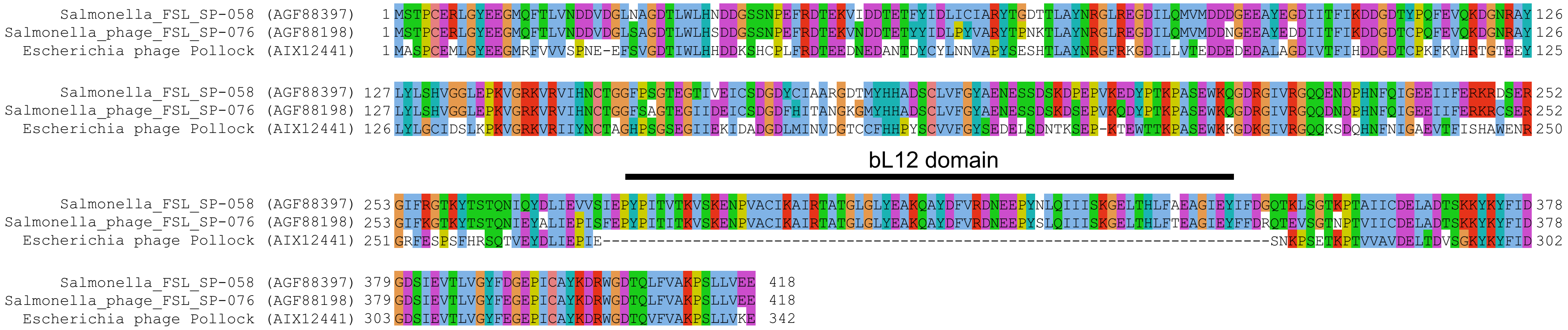

Supplementary Figure 5. Multiple sequence alignment of the bL12 domain containing protein found in Salmonella phages FSL-SP-058 and FSL-SP-076 with the homologous protein encoded by Escherichia phage Pollock. The position of bL12 domain is identified by a black like.

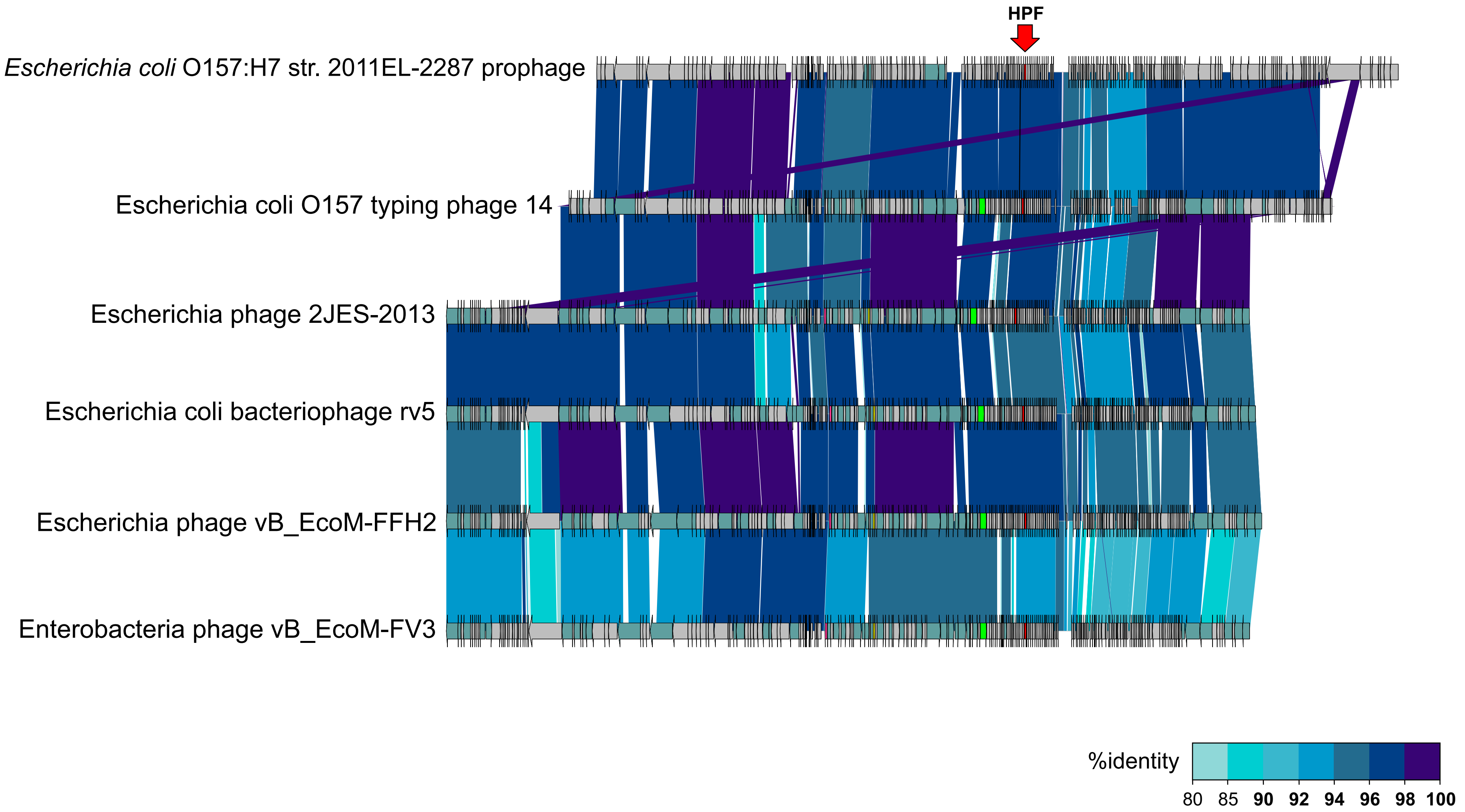

Supplementary Figure 6. Genome comparison of related viral genomes carrying ribosomal protein HPF (S30AE). All comparisons were done at the nucleotide level. A color scale for the % identity is shown at the bottom right. The position of the ribosomal protein-encoding genes is indicated by a red arrow.

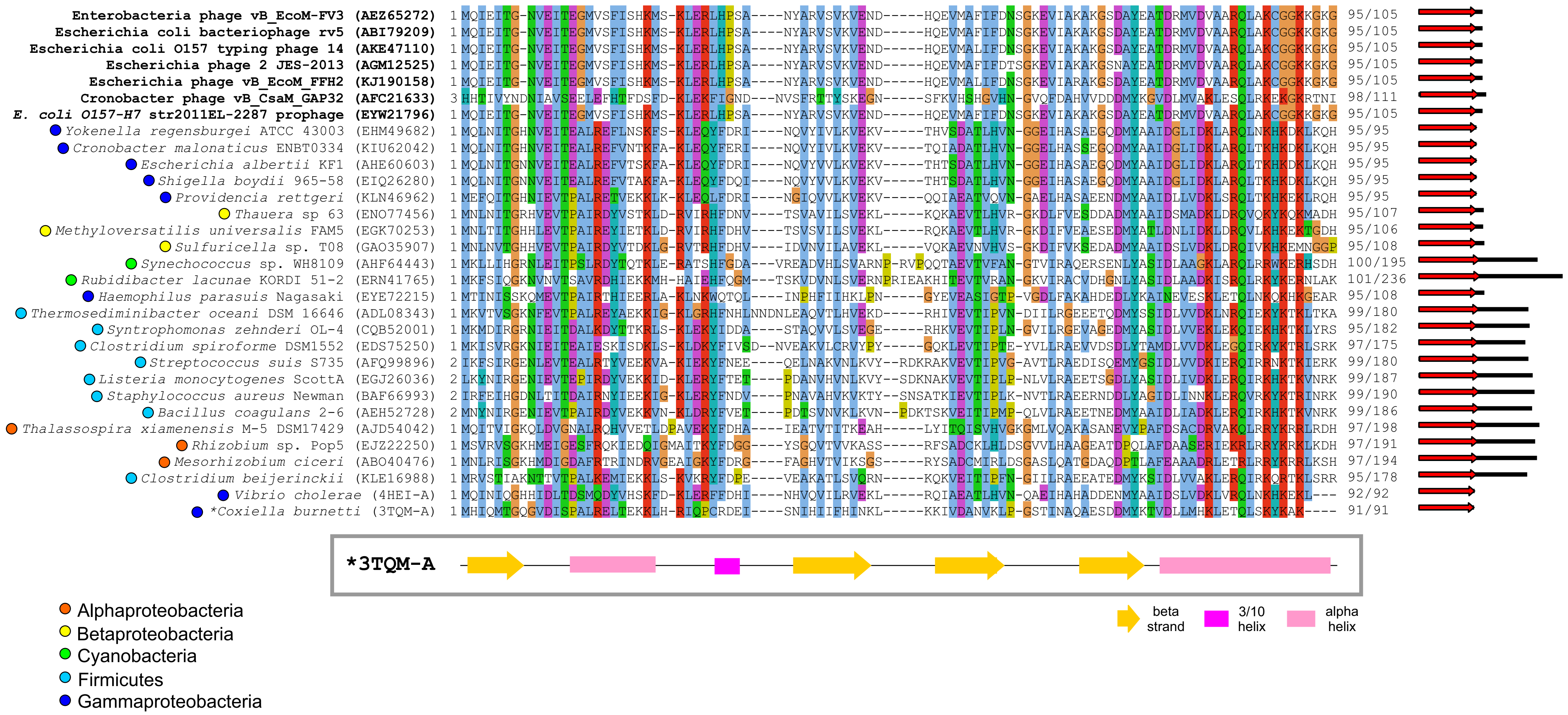

Supplementary Figure 7. Multiple sequence alignment of ribosomal protein HPF (S30AE) identified in six phage genomes with cellular homologs. Taxonomic affiliations are represented by colored circles (see legend at the bottom). Positions of the ribosomal protein domains (red arrows) within the full proteins (black line) are shown at the right side of the alignment.

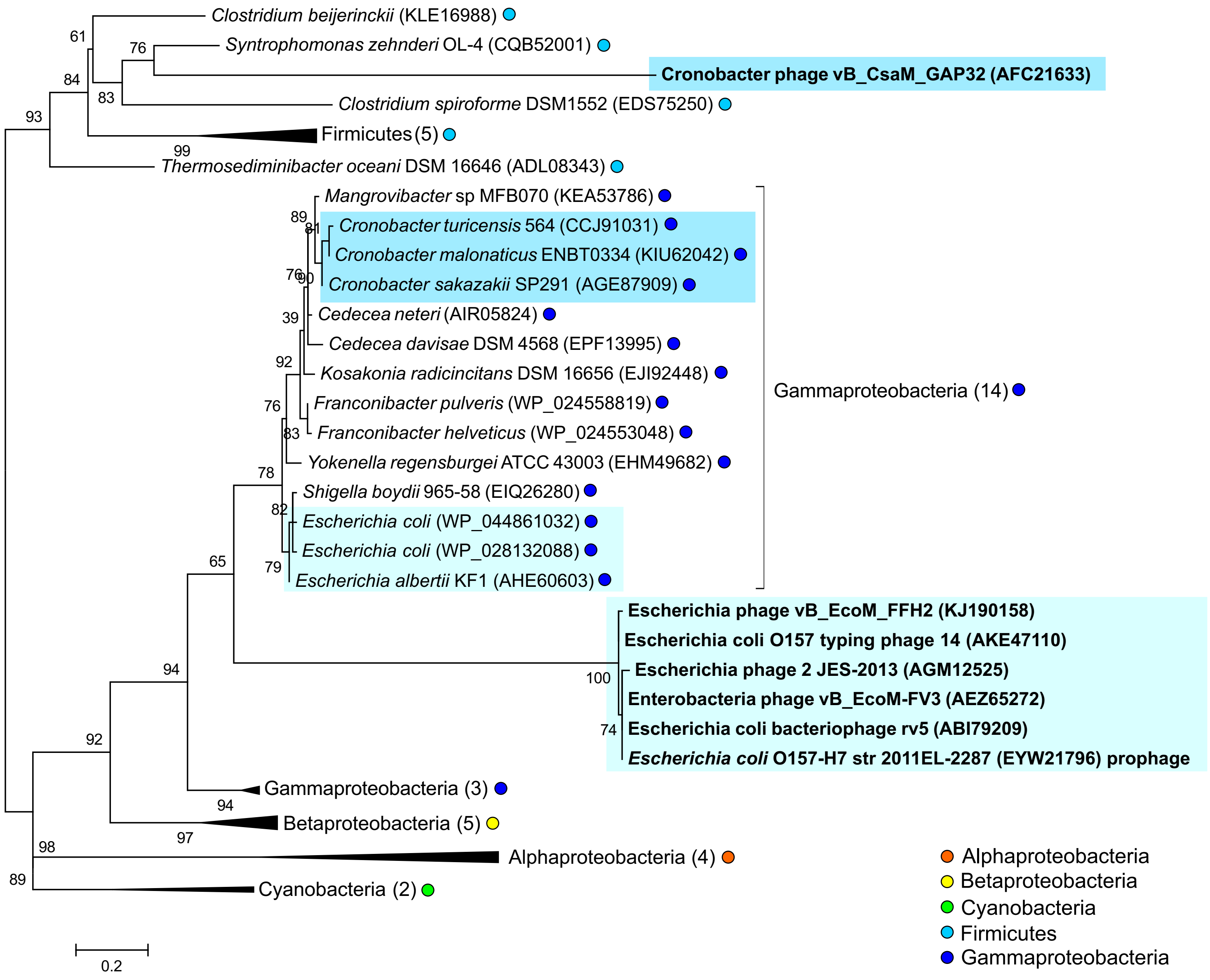

Supplementary Figure 8. Maximum likelihood phylogenetic tree of ribosomal protein HPF (S30AE). Nodes corresponding to viral sequences are shown in bold. Both viruses and hosts are highlighted with the same color. Taxonomic affiliations are represented by colored circles (see legend at the bottom).

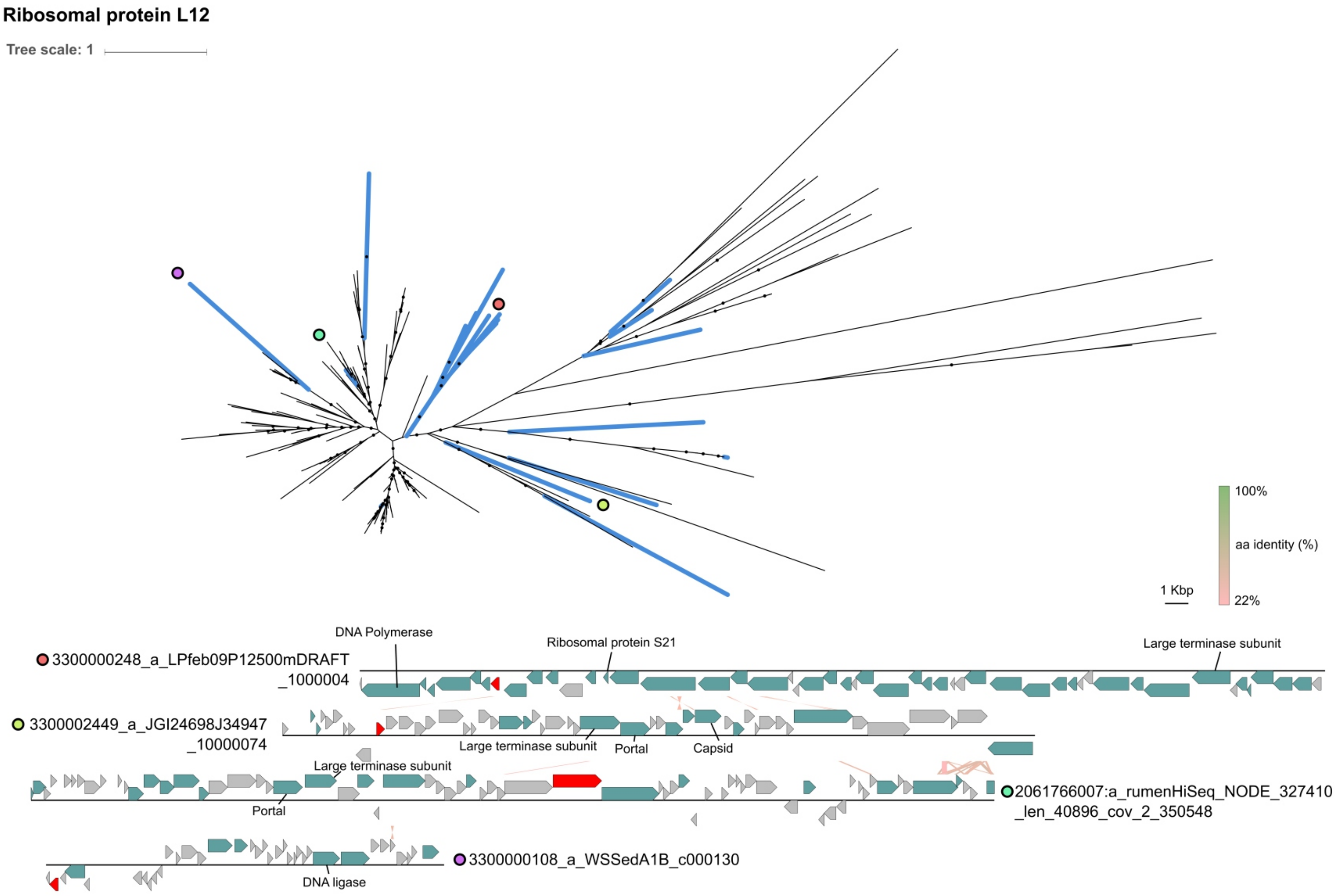

Supplementary Figure 9. Ribosomal proteins bL12 identified in uncultivated viral genomes. Top: Phylogenetic tree of ribosomal protein bL12. Viral clades are highlighted with blue branches. All branches with support < 50% were collapsed, and branches with support ≥ 80% are noted with a black dot. The position of the sequences presented in the bottom panel is shown with colored circles. Bottom: Genome comparison of viral contigs encoding ribosomal protein bL12. Comparisons were done at the amino acid level, with the %identity displayed with a color scale. The predicted gene encoding ribosomal protein bL12 is indicated in red.

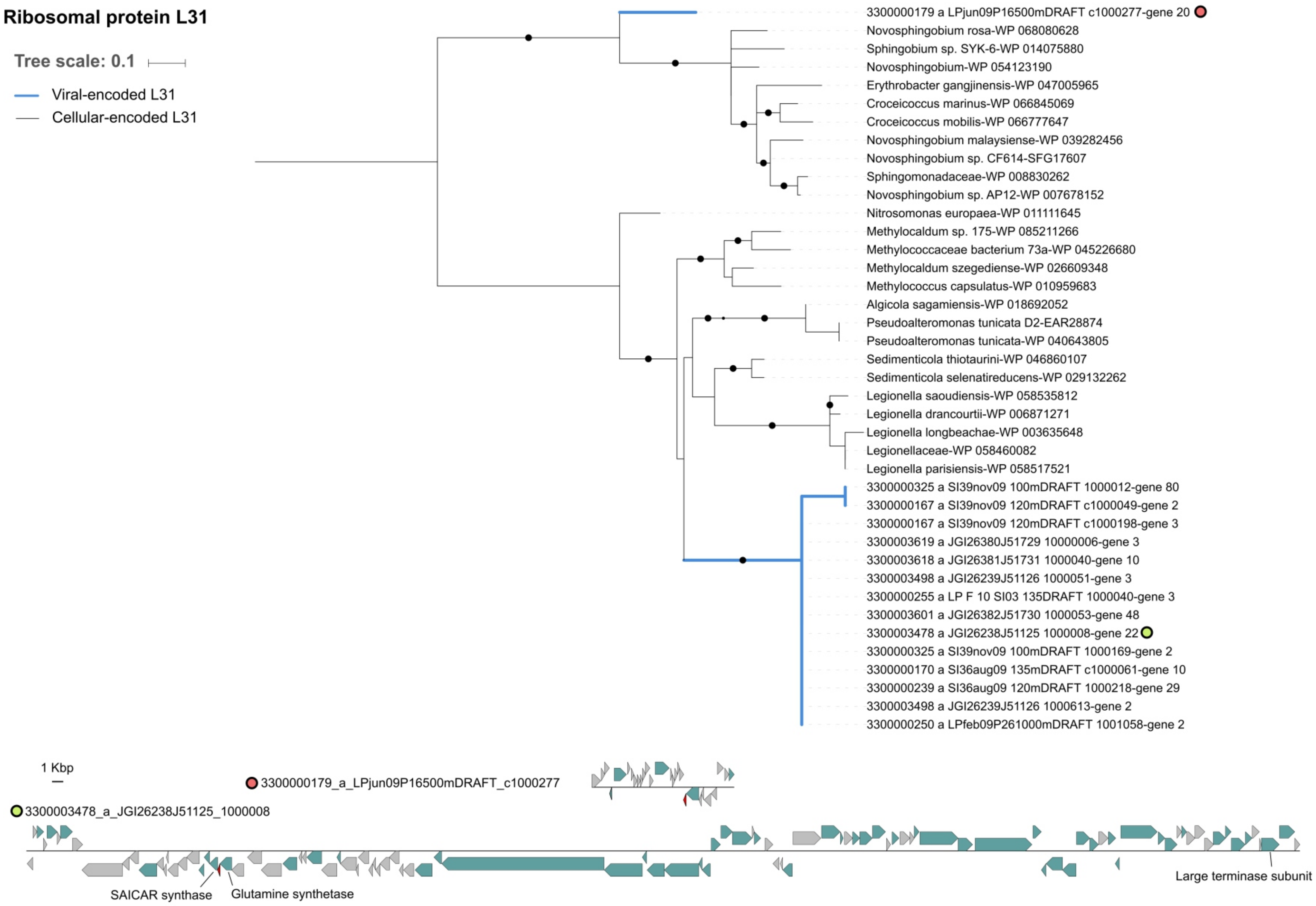

Supplementary Figure 10. Ribosomal proteins bL31 identified in uncultivated viral genomes. Top: Phylogenetic tree of ribosomal protein bL31. Viral clades are highlighted with blue branches. All branches with support < 50% were collapsed, and branches with support ≥ 80% are noted with a black dot. The position of the sequences presented in the bottom panel is shown with colored circles. Bottom: Genome maps of viral contigs encoding ribosomal protein bL31. The predicted gene encoding ribosomal protein bL31 is indicated in red.

Ribosomal protein L33

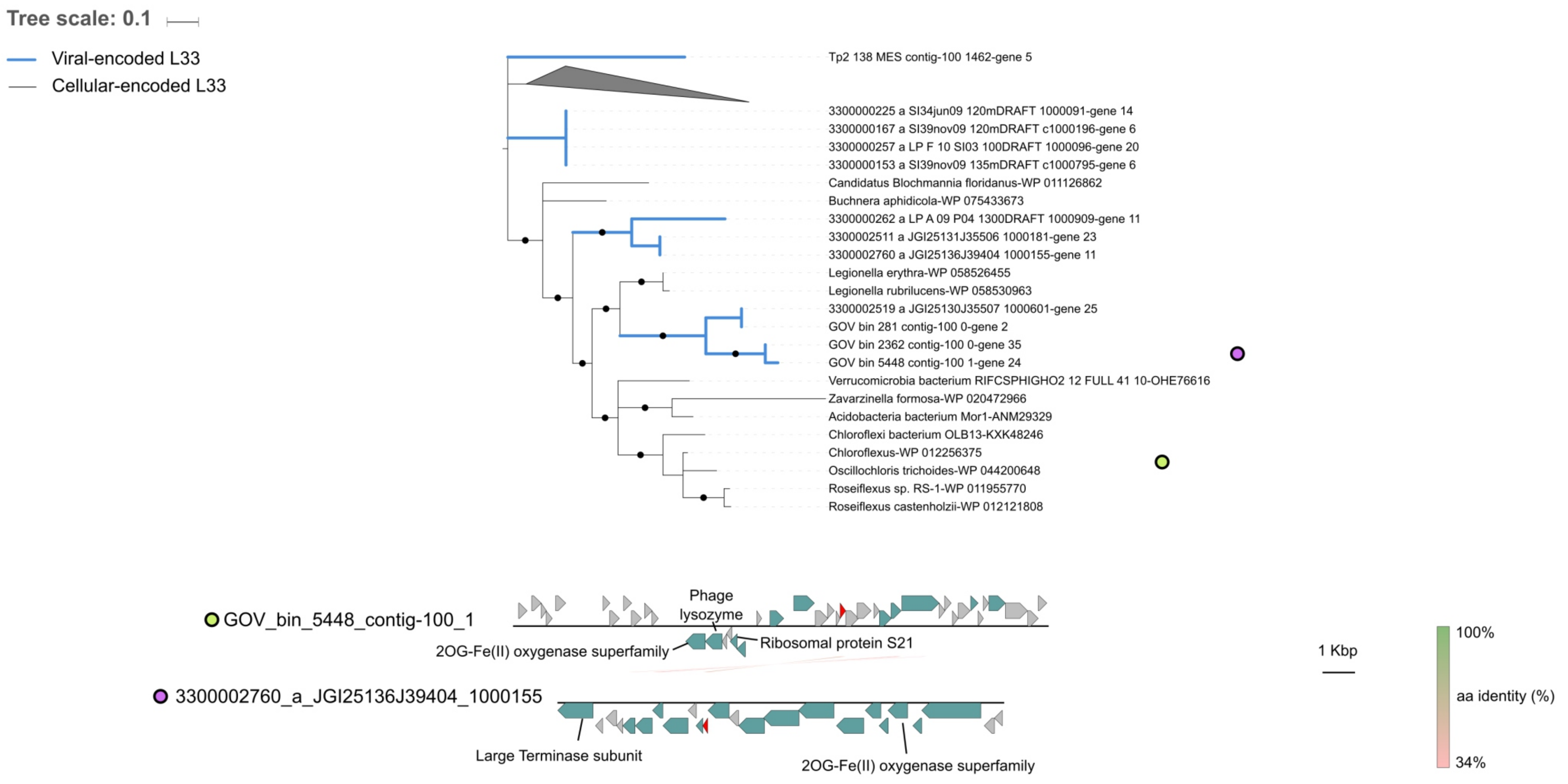

Supplementary Figure 11. Ribosomal proteins bL33 identified in uncultivated viral genomes. Top: Phylogenetic tree of ribosomal protein bL33. Viral clades are highlighted with blue branches. All branches with support < 50% were collapsed, and branches with support ≥ 80% are noted with a black dot. The position of the sequences presented in the bottom panel is shown with colored circles. Bottom: Genome comparison of viral contigs encoding ribosomal protein bL33. Comparisons were done at the amino acid level, with the %identity displayed with a color scale. The predicted gene encoding ribosomal protein bL33 is indicated in red.

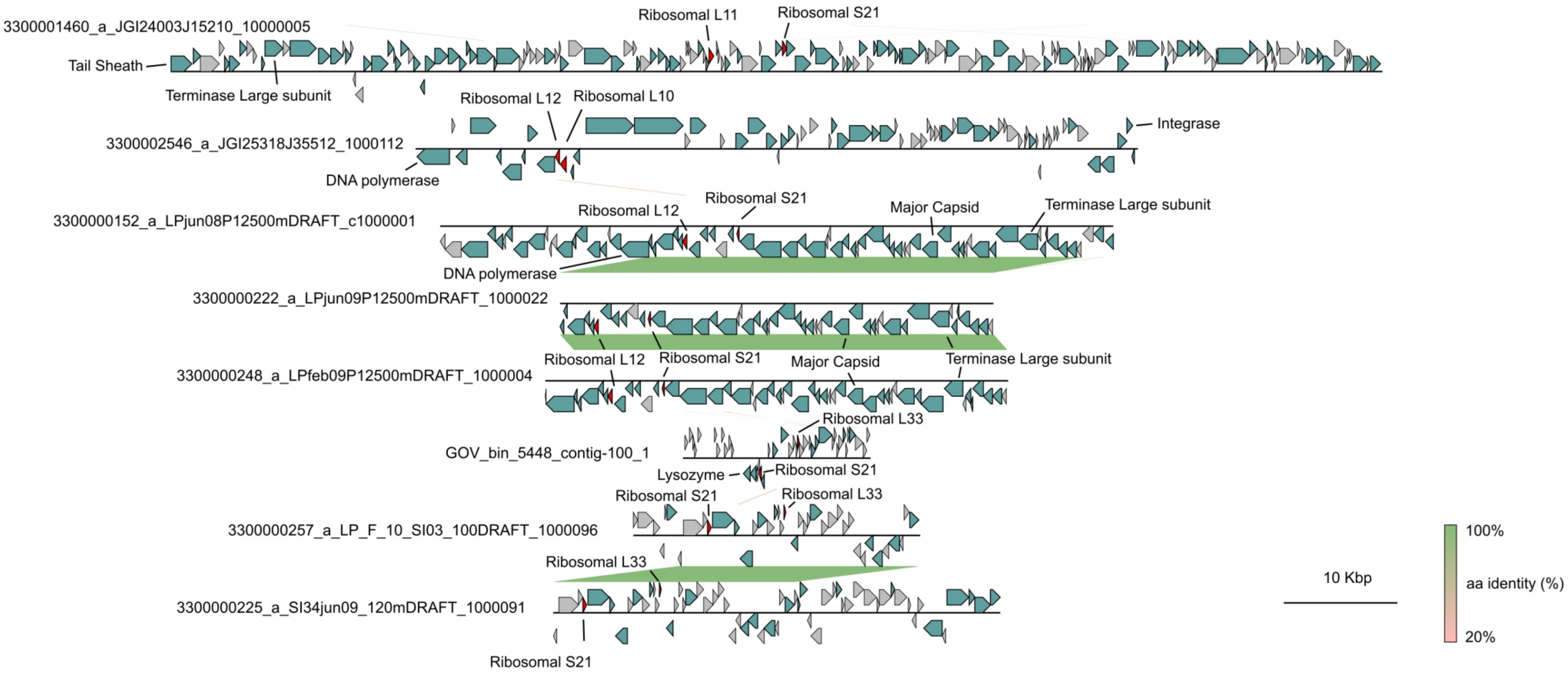

Supplementary Figure 12. Genome map comparison of uncultivated viruses encoding distinct ribosomal proteins. Comparisons were done at the amino acid level, with the %identity displayed with a color scale. Predicted genes encoding ribosomal proteins are indicated in red.

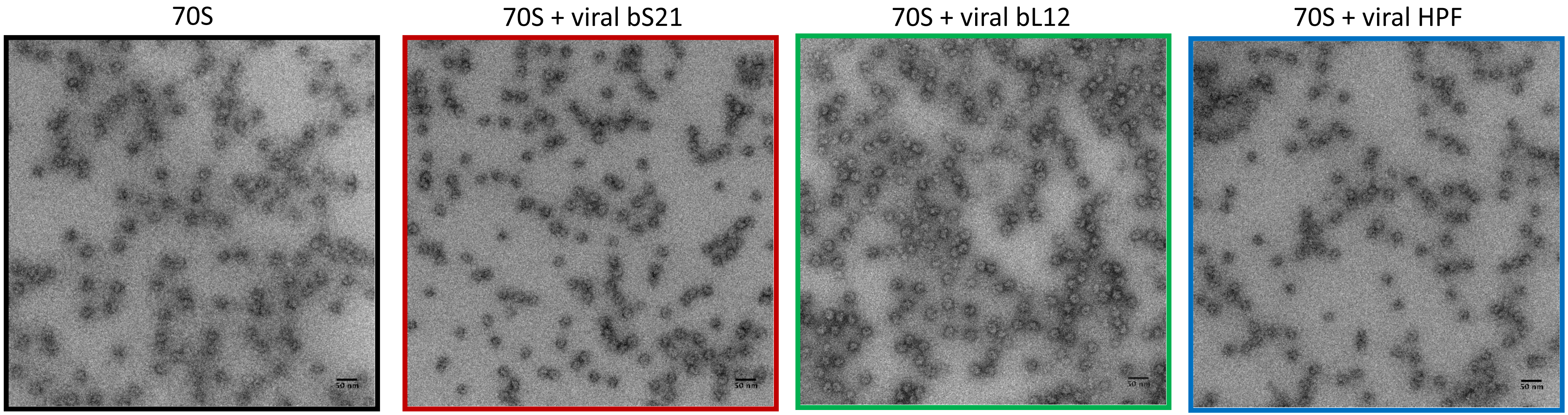

Supplementary Figure 13. Verification of the effect of viral protein expression on ribosome stability. Electron micrographs show negatively stained 70S ribosomes purified from either wilde type NM522 *E. coli* cells (black) or NM522 *E. coli* expressing viral bS21 (red), bL12 (green) or HPF (blue). Scale bars: 50 nm.
