# Supplementary material for: Numerous cultivated and uncultivated viruses encode ribosomal proteins
Source: Nat Commun. 2019 Feb 14;10:752. doi: 10.1038/s41467-019-08672-6 (PMC6375957; doi:10.1038/s41467-019-08672-6)
Supplement: Supplementary file 3 — Description of Additional Supplementary Files [file 41467_2019_8672_MOESM3_ESM.pdf]

Description of Additional Supplementary Files

File Name: Supplementary Data 1
Description: Hosts predictions for 74 uncultivated viruses encoding ribosomal proteins.

File Name: Supplementary Data 2
Description: List of 54 proteins identified in ribosomes purified from E.coli cells after expression of viral bS21 protein, including detailed mass spectrometry information on peptide sequences. The table also includes extended information on phage protein identification in bS21 ribosomal sample.

File Name: Supplementary Data 3
Description: List of 80 proteins identified in ribosomes purified from E.coli cells after expression of viral bL12 protein, including detailed mass spectrometry information on peptide sequences. The table also includes extended information on phage protein identification in bL12 ribosomal sample.

File Name: Supplementary Data 4
Description: List of 71 proteins identified in ribosomes purified from E.coli cells after expression of viral HPF protein, including detailed mass spectrometry information on peptide sequenced. Beta-galactosidase was added as an internal control.

File Name: Supplementary Data 5
Description: List of 279 proteins identified in crude E.coli cell extracts after expression of viral HPF protein, including detailed mass spectrometry information on peptide sequenced.
